# Congenital cytomegalovirus retinitis of prematurity: a case report and literature review

**DOI:** 10.3389/fped.2025.1558820

**Published:** 2025-04-03

**Authors:** Yihui Li, Wenqiang Sun, Xinyun Jin, Lei Zhao, Xueping Zhu

**Affiliations:** ^1^ Department of Neonatology, Children’s Hospital of Soochow University, Suzhou, China; ^2^ Department of Ophthalmology, Children's Hospital of Soochow University, Suzhou, China

**Keywords:** congenital, cytomegalovirus infection, retinitis, prematurity, ganciclovir

## Abstract

**Introduction:**

Ophthalmopathy induced by cytomegalovirus (CMV) infection is most common in immunodeficient patients without other congenital infections. This paper reports a clinical case of retinitis due to congenital CMV infection in a preterm infant and reviews the relevant literature.

**Case presentation:**

A 2-day-old female infant at 36^+2^ gestation weeks presented with a 2-day history of scattered bleeding spots across the body, hemorrhagic diathesis, thrombocytopenia, positive blood CMV IgM, and blood and urine CMV DNA levels significantly above the detection limit by PCR analysis. Maternal serological examination indicated blood CMV IgM positivity. The laboratory test results, CMV IgM positivity in the mother's blood was used to confirm a diagnosis of congenital CMV infection. Later, antiviral treatment with ganciclovir was provided for 3 weeks. Fundus examination indicated a few white exudates along the peripheral retina in both eyes, with a white sheath of peripheral retinal vessels above the temporal plane in the right eye. A diagnosis of CMV retinitis was considered after obtaining abnormal fluorescein fundus angiography results. Ganciclovir was administered at 0.5 mg weekly into the vitreous cavities of the eyes for 3 weeks, and the vascular white sheaths disappeared. Retinitis recurred at 6 months of age, and antiviral treatment was recommended. However, the family rejected it.

**Conclusion:**

Congenital cytomegalovirus retinitis carries substantial risks. For infants suspected of this condition, early initiation of antiviral therapy is crucial to enable timely intervention, improve prognosis, and enhance the child's quality of life.

## 1 Introduction

Human cytomegalovirus (HCMV) is a DNA virus from the *Herpesviridae* family that only infects humans. It is mainly transmitted through the placenta, breastfeeding, sexual contact, blood transfusion, solid organ transplantation, or hematopoietic stem cell transplantation (1). HCMV is a major cause of congenital infections worldwide, with a prevalence of nearly 0.2%–6.1% in live births (2).

Cytomegalovirus (CMV) infections can present with ocular involvement, severely affecting visual function, and ocular abnormalities manifesting as retinitis, corneal endotheliitis, and anterior uveitis, with CMV retinitis being the most common (3). CMV retinitis is observed in patients with immunodeficiencies, such as acquired immunodeficiency syndrome (1). However, its onset in preterm infants with congenital CMV infections is rare. This article analyzes the clinical data of a preterm infant with congenital CMV infection associated with retinitis. The clinical characteristics, diagnosis, treatment, and prognosis of retinitis in preterm infants with CMV infections are discussed to improve early recognition and overall disease outcomes.

## 2 Methods

This study retrospectively analyzed the clinical data of a preterm infant with ocular involvement after congenital CMV infection admitted to the neonatal ward of the Affiliated Children's Hospital of Soochow University. The Ethics Committee of the Children's Hospital of Soochow University approved the study. Written informed consent was obtained from the patient's family.

In the literature review, the keywords “congenital,” “cytomegalovirus retinitis,” “newborn,” and “infant” were used to search foreign databases, such as PubMed and Elsevier, from database inception to April 2024. Immunocompromised patients were excluded. Articles were screened according to the following criteria: English language and full-text availability, and whether the search involved articles on clinical manifestations, diagnostic tests, or treatments of patients. This study was a single case report and the literature review and was not designed as a systematic review. Therefore, its methodological quality could not be assessed.

## 3 Case presentation

A 2-day-old female infant at 36 + 2 weeks of gestation presented with tachypnea present since birth and scattered petechiae over the body for 2 days, accompanied by hemorrhagic diathesis, thrombocytopenia, positive CMV IgM serology, and high CMV DNA viral loads exceeding the detection limit in both blood and urine specimens by PCR analysis. The child was the result of the mother's first pregnancy and first delivery (G1P1), delivered by cesarean section in an outside hospital because of reduced blood flow in the umbilical artery at a gestational age of 36 ± 2 weeks, with a birth weight of 2,750 g. The mother's screening test was positive for CMV IgM. However, she did not receive treatment and had no other complications during her pregnancy. She had one episode of elevated blood glucose, which became normal after dietary modifications. The child experienced shortness of breath and exhibited poor crying post-birth. This was accompanied by slight face and limb bruising and scattered pinpoint-sized hemorrhages on the trunk and facial skin. After admission, paired blood culture sets (two bottles per set) were conducted, and the results were negative after 5 days of incubation. The blood was positive for CMV IgM. Chest x-ray showed thickening and blurring of the lung texture, and patchy or diffuse exudative shadows were seen, suggesting inflammation in both lungs. The patient was given hooded oxygen, cephalosporin, adenosine monophosphate, and intravenous gammaglobulin infusion. For further management, the patient was transferred to our hospital's Neonatal Intensive Care Unit. On admission, the examination revealed a temperature (T) of 36.5℃, pulse (P) of 135 beats/min, respiration (R) of 65 breaths/min, weight (Wt) of 2.65 kg, and percutaneous arterial oxygen saturation (SPO_2_) of 94% (box oxygen). The patient demonstrated poor mental response, irritability, and occasional limb tremors. Scattered petechiae could be seen on the skin, with ecchymosis observed around the mouth and feet. The skin appeared dry, and pinpoint-sized bleeding spots gradually spread across the body. The fontanelle was flat and soft, about 1.0 cm × 1.0 cm. The neck was soft, and slight shortness of breath was observed because of the inspiratory triple concave sign. Neither dry nor wet rales were heard, the rhythm was synchronous, the precordial heart rhythm was regular, and the heart sounds were moderate. However, a grade 2/6 murmur was heard in the precordial region. The abdomen was dilated, the liver was 3 cm below the ribs, the spleen could not be detected below the ribs, the intestinal sounds were audible, the limb muscle tone was slightly high, the limb ends were warm, and primitive reflexes were poorly elicited.

Upon admission, CMV and hearing examinations indicated left and right hearing thresholds at 100 dB Sound Pressure Level. No abnormalities were seen in the ophthalmological screening for retinopathy of prematurity. The patient was treated with latamoxef (40 mg/kg, twice daily) for infection, along with filtered white platelets (0.5 units) to prevent hemorrhage and compound glycyrrhizin combined with adenosine monophosphate for liver protection. Additionally, intravenous ganciclovir was administered (5 mg/kg/dose every 12 h for 2 weeks). Maintenance treatment was provided for a week, during which the ophthalmological examination showed no significant abnormalities. Cranial magnetic resonance imaging revealed bilateral lateral ventricle hemorrhage, subarachnoid hemorrhage, and brain alterations characteristic of preterm infants. The spontaneous hemorrhage was likely attributed to thrombocytopenia, and the prior treatment regimen was continued for two additional days. Platelet levels stabilized at 42 × 10^9^/L, skin hemorrhagic spots diminished, liver function normalized, and vital signs were stable. Hence, the patient was transferred to the general ward. In the general ward, the patient received latamoxef (40 mg/kg, twice daily) and ganciclovir (5 mg/kg/dose, every 12 h). The ganciclovir dose was reduced to 5 mg/kg/d followed a 17-day induction. CMV DNA viral loads were quantified by quantitative PCR in paired urine and plasma specimens. The fundoscopic examination indicated stage I retinopathy in zone III of the right eye. Antiviral maintenance therapy was continued, and subsequent ophthalmological screening established stage I retinopathy within the same region. Another CMV examination was repeated, followed by the discontinuation of ganciclovir.

**Figure 1:**
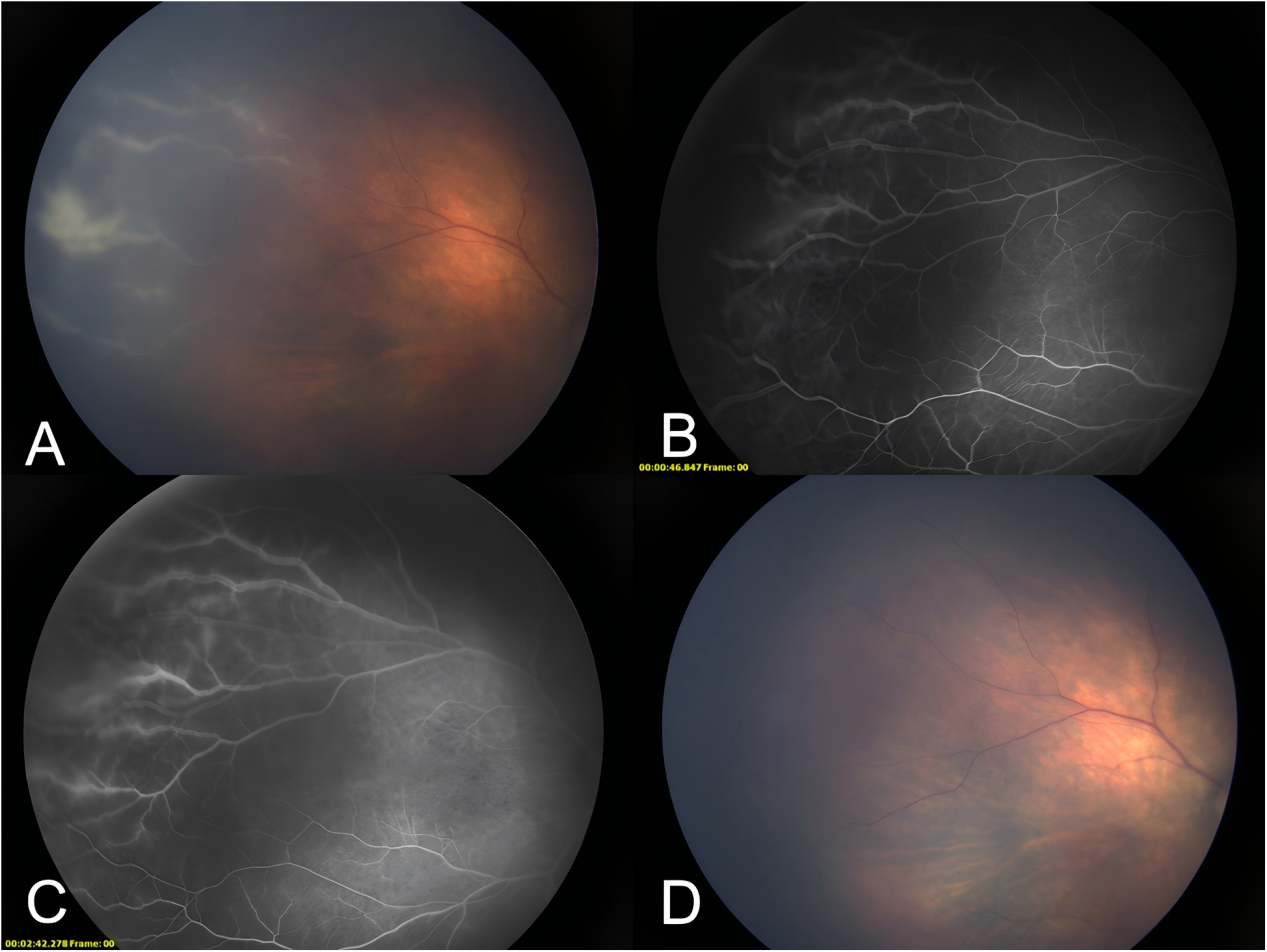
A photograph of the fundus of the patient. **(A)** The white sheath of peripheral retinal vessels above the temporal plane of the right eye. **(B)** Early blurring of peripheral retinal vessels above the temporal plane of the right eye, with fluorescein leakage. **(C)** Late stage of the peripheral retinal vessels above the temporal plane of the right eye with worsening fluorescein leakage. **(D)** The white sheath of peripheral retinal vessels above the temporal plane of the right eye disappeared after three treatments with vitreous cavity ganciclovir injections.

Following 34 days of therapeutic intervention, the pediatric patient was discharged in an afebrile state with intact mental status and age-appropriate responsiveness. No significant bleeding spots were observed on the skin or mucous membranes. The liver was 2 cm below the ribs, with the spleen not palpable. The platelet count was recorded at 177 × 10^9^/L. Five weeks after birth, the patient underwent a follow-up fundus examination in the ophthalmology clinic of our hospital. There was a slight white exudation in the vascular distribution of the peripheral retina in both eyes and a white sheath of the peripheral retinal vessels above the temporal surface of the right eye (Figure 1A). Fundus fluorescence angiography indicated blurred edges and fluorescein leakage in the peripheral retinal vessels at an early stage above the temporal plane of the right eye (Figure 1B), which worsened later (Figure 1C). A positive CMV DNA test or positive rapid virus isolation in urine, saliva, and/or blood samples within the first 3 weeks of life indicates congenital infection. In contrast, if the pathogen detection results are negative during the first 3 weeks after birth but become positive for CMV DNA or rapid virus isolation in urine, saliva, and/or blood samples after 3 weeks, this suggests an acquired infection (4). Based on the child's medical history and the clinical data, the ocular lesions were identified as CMV retinitis. Weekly injections of 0.5 mg ganciclovir were given to both eyes for 3 weeks, inducing the disappearance of the vascular white sheaths (Figure 1D). Retinitis recurred at 6 months of age, and the patient was followed up by telephone. However, the parents were not compliant and voluntarily abandoned the treatment. This case included routine blood examinations (Table 1) and CMV load results (Table 2).

**Table 1:** The child's laboratory blood test results.

Date	1.19	1.21	1.25	1.28	1.29	1.31	2.03	2.12	2.16	2.20
WBC count (×10^9^/L)	7.99	7.35	10.35	6.91	9.5	6.45	7.51	–	8.48	13.73
LY percentage (%)	72.1	69.8	74.6	84.9	87.1	85.7	76.7	–	87.0	61.5
HB (g/L)	172	152	154	140	141	132	121	–	96	92
RBC count (×10^12^/L)	5.04	4.51	4.72	4.38	4.37	3.94	3.72	–	3.56	2.89
PLT count (×10^9^/L)	15	116	41	42	45	63	121	–	177	225
NE percentage (%)	17.4	11.7	16.5	11.2	9.2	11.4	22.1	–	12.6	26.6
CRP (mg/L)	23.94	3.17	1.58	1.32	0.86	0.62	1.25	–	0.67	–
ALT (U/L)	10.5	–	52.2	34.7	–	–	32.9	159.4	92.4	63.8
AST (U/L)	132.1	–	172.3	87.4	–	–	62.2	180.3	75.3	59.4
LDH (U/L)	1,563.7	–	669.1	621.7	–	–	363.5	411.9	266.5	414.1
TBIL (μmol/L)	64.7	–	21.8	16.1	–	–	10.1	6.9	6.3	4.7
DBIL (μmol/L)	20.8	–	10.95	8.64	–	–	6.3	4.4	3.64	2.5
IBIL (μmol/L)	43.9	–	10.85	7.46	–	–	3.8	2.5	2.66	2.2
GGT (U/L)	89.1	–	358.9	462.3	–	–	502.1	289	216.5	146.9
ALP (U/L)	363.7	–	228	241	–	–	247	257	268	267.7
TP (g/L)	75.4	–	65.5	64.1	–	–	61.2	57.2	58	62.6
ALB (g/L)	32.3	–	31.1	31	–	–	31.4	32.1	33.9	36.4

WBC, white blood cell; LY, lymphocyte; HB, hemoglobin; RBC, red blood cell; PLT, platelet; NE, neutrophil; CRP, C-reactive protein; ALT, alanine aminotransferase; AST, aspartate aminotransferase; LDH, lactate dehydrogenase; TBIIL, total bilirubin; DBIL, direct bilirubin; IBIL, indirect bilirubin; GGT, gamma-glutamyltransferase; ALP, alkaline phosphatase; TP, total protein; ALB, albumin.

**Table 2:** Cytomegalovirus laboratory findings in the patient.

Date	Cytomegalovirus DNA (urine)	Cytomegalovirus DNA (blood)
Day 3 of life	5.27 × 10^7^ copies/ml	2.57 × 10^4^ copies/ml
Day 21 of life	2.93 × 10^7^ copies/ml	1.40 × 10^3^ copies/ml
Day 36 of life	2.41 × 10^7^ copies/ml	1.45 × 10^3^ copies/ml

## 4 Literature review and discussion

CMV retinitis occurs in immunocompromised patients. However, disease onset in premature infants with congenital CMV infections has been rarely reported. Congenital CMV infection is associated with a high mortality rate, and survivors tend to recover from non-neurological damage. However, neurological damage can be irreversible. The incidence is high and damaging in preterm infants. Moreover, congenital CMV infection affects multiple organs in the body. Still, no clear treatment guidelines have been developed, and adult treatment protocols are used. The current literature search identified 11 relevant papers involving 11 children with incomplete case information. A summary of the reported cases is represented in Table 3 (5–15). Gestational age was recorded in 10 cases, among which six were preterm. However, the early diagnosis and treatment of preterm infants did not focus on congenital CMV retinitis, resulting in untimely treatment. Therefore, preterm infants with maternal CMV and a CMV infection history must undergo a timely ophthalmological assessment to exclude this pathology.

**Table 3:** Clinical characteristics of 12 patients with congenital cytomegalovirus retinitis (including our case).

Patient	Gender	Gestational age	Onset time	Diagnostic methods	Serum CMV antibodies in the mother	Clinical manifestation	Treatment	Outcome
Patient 1	Female	37 weeks	At birth	Serum CMV IgM-positive + urine CMV isolation + clinical features (ventriculomegaly and thrombocytopenia)	Positive CMV IgG and negative IgM in late pregnancy serum	Thrombocytopenia, hearing impairment, hepatitis, hepatomegaly, and uveitis	Intravenous ganciclovir (5 mg/kg three times daily for 3 weeks) + CMV immunoglobulin	Platelet count recovered, enlarged liver and spleen/ventricles resolved, and neuromotor activity was normal after 5 months
Patient 2	Female	41 weeks	Four weeks of age	Elevated CMV IgG/IgM + blood/urine CMV DNA positivity (PCR)	The titers of CMV IgG and IgM were elevated	Hepatitis, retinitis, and protein-losing gastrointestinal disease	intravenous ganciclovir (5 mg/kg twice daily for 2 weeks) + CMV immunoglobulin (2 weeks)	Diarrhea and uveitis resolved, and urinary CMV DNA remained positive at 8 months but without sequelae
Patient 3	Unknown	35 weeks	One day after birth	Urine CMV culture + PCR positive	The antibody status of the mother was not clearly described	Retinitis, microcephaly, and intracranial calcification	Intravitreal ganciclovir sodium injection (12 times)	Retinitis improved briefly but there was a recurrence without systemic complications
Patient 4	Male	unknown	Seven months of age	Blood/urine CMV DNA high load (PCR) + serology (hypergammaglobulinemia)	The antibody status of the mother was not clearly described	Hepatomegaly, retinitis with dystrophic intraocular calcification, and bone marrow suppression	Ganciclovir (5 mg/kg/day) + IVIG (discontinued because of neutropenia), followed by uncontrolled recurrence	Died of disseminated CMV infection (respiratory failure)
Patient 5	Female	32 weeks	19th day of life	Maternal serum CMV positive + infant urine CMV positive + fundus examination (frosted glass vitreitis)	IgG was positive, IgM was negative	Retinitis	Intravenous ganciclovir (6 mg/kg/day for 42 days) + intravitreal foscarnet injection	Retinitis resolved completely after repeated episodes, leaving a macular scar
Patient 6	Female	36 weeks	4th day of life	Aqueous humor CMV DNA positive (PCR) + serology (maternal CMV IgM positive)	Both CMV IgG and IgM were positive	Hepatomegaly, retinitis, microcephaly	Oral valganciclovir (6 weeks)	Vasodilation subsided, leaving choroidal retinal atrophy
Patient 7	Unknown	36 weeks	Present from birth	Vitreous/serum CMV DNA positive + UL97 gene testing (ganciclovir resistance)	The antibody status of the mother was not clearly described	Retinitis	Ganciclovir (6 mg/kg twice daily for 62 days) + foscarnet + laser photocoagulation	Viral load decreased and retinitis subsided, followed by oral valganciclovir prophylaxis
Patient 8	Male	37 weeks	Four months of age	Aqueous humor/cerebrospinal fluid CMV DNA positive (PCR) + maternal serum IgG positive (IgM negative)	CMV IgG was positive and IgM was negative	Retinitis and central nervous system involvement	Intravenous ganciclovir (10 mg/kg/day) + intravitreal injection (1 mg/0.1 cc twice weekly)	Death from CMV encephalitis (central nervous system involvement)
Patient 9	Unknown	26 weeks	Gestational age 39 weeks	Serum CMV IgM/IgG positive + urine CMV DNA (PCR)	The antibody status of the mother was not clearly described	Thrombocytopenia and retinitis	FA-guided laser treatment for ROP + oral valganciclovir (5 ml daily)	Retinal inflammation healed and vision normalized
Patient 10	Female	unknown	Four weeks of age	Serum CMV IgM/IgG positive + confirmed by PCR	The antibody status of the mother was not clearly described	Retinitis	Intravenous ganciclovir (10 mg/kg/day for 4 weeks) → oral valganciclovir (4 weeks)	Choroidal retinitis healed and general condition improved
Patient 11	Unknown	38 weeks	One day after birth	Urine CMV DNA high viral load (PCR) + UL97 gene mutation (A594P, ganciclovir resistant)	CMV IgM was positive, but IgG was not mentioned	Thrombocytopenia, hearing impairment, microcephaly, uveitis	Ganciclovir (5 mg/kg every 12 h for 16 days) + phosphonoformate combination → oral valganciclovir	Retinitis subsided and CMV viral load stabilized
Patient 12	Female	36 weeks	One day after birth	Mother CMV IgM-positive + infant blood/urine CMV DNA-positive (PCR) + fundus examination (vascular pallor and fluorescein leakage)	CMV IgM was positive, but IgG was not mentioned.	Thrombocytopenia, hearing impairment, hepatitis, hepatomegaly, and retinitis	Intravenous ganciclovir (17 days) → intravitreal ganciclovir injection (3 weeks)	Treatment was abandoned after retinitis recurrence, and the disease was uncontrolled at 6 months

CMV, cytomegalovirus; PCR, polymerase chain reaction; IVIG, intravenous immunoglobulin; IgG, immunoglobulin G; IgM, immunoglobulin M.

Our patient (Case 12) is consistent with previously reported congenital CMV retinitis cases in core diagnostic methods (including serological IgM positivity and blood or urine CMV DNA PCR testing) and first-line treatment strategies (intravenous ganciclovir combined with intravitreal injection). Both cases face challenges of retinitis recurrence. The subsequent use of intravitreal injections leads to high drug concentrations in the retina and vitreous cavity, suppressing viral replication and controlling local infection. This prevents progression to the macula and irreversible vision loss. We did not perform drug resistance gene testing (e.g., UL97/UL54 mutation analysis) because of the risk of systemic toxicity and the patient's existing thrombocytopenia, delaying treatment adjustment. Additionally, poor family adherence (abandoning treatment post-recurrence) directly contributed to disease deterioration. This was in contrast with most cases in which the prognosis improved from the use of foscarnet, valganciclovir, or laser interventions. Unlike typical intrauterine infection cases (e.g., Cases 1, 5, and 6), this case may depict a perinatal infection (later mother-to-child transmission), similar to Case 8, but without fatal involvement of the central nervous system. In this case, the MRI findings of intraventricular hemorrhage in the preterm infant, potentially associated with placental hypoperfusion and perinatal asphyxia, suggest that MRI serves as a promising tool for detecting fetal brain injury (16). Despite no reported deaths, the poor prognosis underscores the crucial role of resistance monitoring, long-term follow-up, and parental education while managing CMV retinitis. This case also highlights the multisystem involvement characteristic of perinatal asphyxia, emphasizing the importance of combining early imaging with biomarker analysis for comprehensive evaluation (17–19).

CMV infection of retinal pericytes primarily presents as a lytic infection. It damages the proximal region of the inner blood-retinal barrier (IBRB), significantly losing these supportive cells (20). In premature infants, the structural integrity of the IBRB is significantly decreased because of vascular dysplasia (e.g., persistent avascular zones) (21). This facilitates the hematogenous dissemination of CMV into retinal tissue. Studies have demonstrated that hereditary NOS2 gene homozygous frameshift mutations, resulting in loss of nitric oxide synthase 2 function, significantly impair anti-CMV immunity and predispose to fatal infections (22). Furthermore, abnormalities in tetrahydrobiopterin (BH4) metabolism may exacerbate oxidative stress and indirectly compromise immune responses, suggesting that genetic mutations modulate CMV susceptibility through distinct pathways (23). Similarly, CMV can become a lifelong latent infection in immunocompromised individuals via immune evasion mechanisms. These infections can be reactivated by external stimuli, leading to a dynamic cycle of “infection-latency-activation,” (24) which explains the recurrence seen in our case. Typical CMV retinitis presentations involve explosive/hemorrhagic (necrosis and hemorrhage resembling “pizza pie” within the posterior pole) and granular forms (granular lesions in the peripheral retina) (25, 26). In this case, the patient exhibited yellow-white granular necrotic lesions with edema and vascular sheathing within the superior temporal peripheral retina (Zone III). This matched the granular lesion characteristics in the literature without hemorrhagic manifestations. The patient presented with thrombocytopenia (15 × 10⁹/L), systemic petechiae, and retinitis (fluorescein leakage and vascular sheathing), which are consistent with typical congenital CMV infection features (e.g., hepatosplenomegaly, thrombocytopenia, and ocular involvement) (27). Microcephaly was not reported; however, the rapid progression and recurrence of retinitis indicate systemic and multi-organ involvement of CMV infections. The literature suggests that about 13% of newborns with congenital CMV infections demonstrate identifiable symptoms at birth. The clinical manifestations in Case 12 support this ratio, highlighting the requirement for screening asymptomatic or atypical cases (e.g., 87% of asymptomatic infected infants in a Spanish study were diagnosed by screening) (28). Regular neonatal CMV screening enhances the detection rates for asymptomatic or mildly symptomatic infections (29). In this case, the mother tested positive for CMV IgM late during her pregnancy but did not receive prenatal intervention. This induced an undetected perinatal infection, causing retinitis. When universal screening of saliva or dried blood spots has been implemented, similar cases were diagnosed earlier, enabling timely antiviral treatment (30). This can reduce the risk of retinitis and neurological sequelae. Retinopathy of prematurity involves abnormal retinal vascular development. It typically presents with vascular tortuosity, neovascularization, and staged progression (I–V) (31) but lacks systemic symptoms. Diagnosis depends on fundus examination and preterm birth history. CMV retinitis is caused by either maternal vertical transmission or immunodeficiency (such as human immunodeficiency virus infection). It is characterized by peripheral retinal necrotic lesions with yellow-white exudates and hemorrhage. Moreover, it is accompanied by multi-system manifestations, including hepatosplenomegaly, thrombocytopenia, or encephalitis (27). The key diagnostic tests involve CMV DNA PCR analysis (from blood, urine, or aqueous humor) or positive CMV IgM antibodies. Due to the potential visual impairment induced by congenital CMV infection, infants having ocular-related clinical manifestations at birth must undergo at least one eye examination every year (32).

Ganciclovir, an antiviral drug approved by the Food and Drug Administration in 1989, has been a cornerstone of CMV retinitis treatment and prevention. It inhibits viral DNA polymerase, effectively blocking CMV replication (33). Ganciclovir is administered systemically or through intravitreal injection. Long-term antiviral therapy has a dual purpose in managing congenital CMV infections. It controls acute infections while potentially reducing the risk of distant recurrences. Vicente et al. revealed that valacyclovir administration during pregnancy significantly improved the proportion of asymptomatic newborns (79.4%). However, 32.35% of these children developed long-term sequelae. This includes sensorineural hearing loss, suggesting that short-term prenatal treatment may not suppress all complications. Therefore, extended treatment or postnatal maintenance therapy is needed (34). Intravitreal ganciclovir injections bypass the blood-retinal barrier, providing therapeutic concentrations directly within the retina and reducing systemic side effects, such as neutropenia. This route has demonstrated enhanced efficacy in treating CMV retinitis. The results were evidenced in Case 3 in this paper, where the retinal vascular sheath was removed entirely from the right eye of the patient after 3 weeks of intravitreal ganciclovir injections. Dheyab et al. observed that long-term oral valganciclovir (≥450 mg twice daily) treatment of CMV-associated anterior uveitis effectively controlled intraocular pressure and inflammation without recurrence within a follow-up period of 12–108 months, signifying the benefits of prolonged high-dose therapy (35). Additionally, Tranos et al. demonstrated that the recurrence of CMV anterior uveitis was closely associated with treatment duration and specific biomarkers. About 61.4% of patients required oral and topical medications for up to 44 months to manage recurrences, highlighting the necessity for individualized and long-term treatment regimens (36). While surgical interventions like vitrectomy with silicone oil injections are typically utilized for advanced complications, such as retinal detachment (37), long-term antiviral therapy may reduce the need for such procedures. In one study, valganciclovir treatment enabled 35 out of 40 patients with uveitis to avoid surgery, with only one case requiring filtering surgery because of endothelial inflammation. This demonstrates the potential of antiviral therapy in preventing structural damage (35). However, further investigation of the role of antiviral therapy in preventing recurrences during the long-term management of congenital CMV infection is needed.

CMV infections in pregnant women are classified into latent and active infections. Pre-pregnancy CMV IgG-positive/IgM-negative status indicates a latent infection, which rarely causes intrauterine infection and does not adversely affect the fetus. In contrast, an active CMV infection during pregnancy is associated with active viral replication and is divided into primary and non-primary infections (such as reactivated and reinfected infections). Research suggests that the risk of vertical transmission is higher during primary infections (38, 39) and increases with gestational age. Moreover, transmission rates reach approximately 75% during late gestation (40). Maternal primary cytomegalovirus infection co-occurring with severe concurrent infections significantly elevates maternal-fetal risks. Multiple infections may synergistically exacerbate placental barrier damage, leading to higher intrauterine transmission rates, fetal multi-organ involvement, and long-term neurodevelopmental impairments (41, 42). Observational cohort studies found that raising immunoglobulin levels could be a promising intervention to prevent mother-to-child transmission of primary infections and significantly decrease this risk (43). Additionally, aggressive antiviral therapy has been shown to reduce vertical transmission by 70% in maternal primary infection during pregnancy or early gestation (44). Gene and vaccine therapy are novel CMV disease treatment methods that are currently being investigated in animal experiments and require further observation for efficacy (40). Due to the lack of an applicable vaccine, susceptible pregnant women must avoid contact with secretions from known patients. If patient contact occurs, disinfection must be done to minimize transmission risk. This reduces the CMV infection rates of pregnant mothers to decrease congenital CMV infection. Several studies have recommended universal neonatal HCMV screening for the early detection of all CMV infants so that children with uveitis and other sequelae can be identified early for swift intervention and better outcomes (45).

Breastfeeding can reduce the risk of retinopathy of prematurity (46), but it is also a primary route through which neonates can contract postnatal CMV infections (47). Studies demonstrated that fresh breast milk from CMV-positive mothers led to a 19% [95% confidence interval (CI): 11%–32%] prevalence of CMV infection in very low birth weight infants, with 10% showing clinical signs and 4% developing sepsis-like syndrome. However, the infection rate dropped to 4.4% (95% CI: 2.4%–8.2%) when frozen breast milk was used, although the severe disease rate was unchanged (48). Extremely low birth weight (ELBW) infants (birth weight <1,000 g) remain at higher risk. A study indicated that 26% of ELBW infants aged 23–26 weeks were infected via breast milk, all exhibiting clinical symptoms (49). The risk of infection correlates strongly with factors like viral load in breast milk, breastfeeding frequency, and infant comorbidities (50). Currently, four primary feeding strategies have been suggested for the preterm infants of CMV-positive mothers: freeze-thawed breast milk, pasteurized breast milk, direct breastfeeding, and formula. Freezing decreases virus viability without eliminating transmission risk (51). Traditional pasteurization (heating at 62.5°C for 30 min) inactivates CMV and destroys the bioactive components of breast milk (52). Short-term pasteurization (heating at 62.5°C for 5 s) partially decreases the infection risk, but transmission remains possible (53). Importantly, maternal immunity (e.g., serum-neutralizing antibodies or lactoferrin in breast milk) does not provide protective immunity for preterm infants (54, 55). Therefore, clinical decisions must weigh the benefits of breast milk against the risk of CMV infection. However, standardized prevention guidelines are still lacking. Prioritizing pasteurized breast milk is recommended for high-risk groups like ELBW infants, and infection indicators must be dynamically monitored for better-individualized management.

## 5 Strengths and limitations

1. Retrospective analysis of the clinical data and regression of congenital cytomegalovirus-infected retinitis in a premature infant.
2. Our literature review involved a thorough search of congenital cytomegalovirus retinitis in preterm infants born before May 30, 2024, in foreign databases, and the results are discussed.
3. Due to the rarity of the disease, the number of cases was limited. Moreover, the patient's family abandoned treatment at a later stage. This restricted us from performing long-term post-treatment follow-ups.

## 6 Conclusion

This case, as well as the literature review, suggest that congenital CMV retinitis, although rare in preterm infants, poses a significant disability risk. The immaturity of the preterm immune system, viral reactivation, and poor treatment compliance are key factors leading to recurrence. Early combined systemic and local antiviral therapy can effectively manage acute infections. Still, vigilance involving drug resistance and the necessity for long-term maintenance therapy is needed. Parental education and dynamic monitoring (such as viral load and fundoscopy assessments) are critical for improving outcomes. Future efforts must focus on developing standardized clinical pathways for congenital CMV infections while exploring prenatal screening and perinatal intervention strategies that can decrease the risks of vertical transmission and long-term sequelae.

## Data Availability

The original contributions presented in the study are included in the article/Supplementary Material, further inquiries can be directed to the corresponding author.
